# Advances in the Application of Direct Injection Mass Spectrometry Techniques to the Analysis of Grape, Wine and Other Alcoholic Beverages

**DOI:** 10.3390/molecules28227642

**Published:** 2023-11-17

**Authors:** Andrea Romano, Vittorio Capozzi, Iuliia Khomenko, Franco Biasioli

**Affiliations:** 1 Research and Innovation Centre, Edmund Mach Foundation, Via Edmund Mach, 1, 38010 San Michele all’Adige, Italy; iuliia.khomenko@fmach.it; 2 Institute of Sciences of Food Production, National Research Council (CNR) of Italy, c/o CS-DAT, 71122 Foggia, Italy; vittorio.capozzi@ispa.cnr.it

**Keywords:** direct injection mass spectrometry, wine, grape, ethanol, volatile organic compounds

## Abstract

Direct injection mass spectrometry (DIMS) entails the direct introduction of a gaseous sample into a mass analyser without prior treatment or separation. DIMS techniques offer the opportunity to monitor processes in time, with limits of detection as low as 0.5 parts per trillion in volume (for a 1 s measurement time) while providing results with high informational content. This review provides insight into current and promising future developments of DIMS in the analysis of grape, wine and other alcoholic beverages. Thanks to its unique characteristics, DIMS allows the online monitoring of volatile organic compounds (VOCs) released by grapes during fermentative bioprocesses or by wine directly from the glass headspace or during drinking. A DIMS-based approach can also be adopted to perform quality control and high-throughput analysis, allowing us to characterise the volatile profile of large sample sets rapidly and in a comprehensive fashion. Furthermore, DIMS presents several characteristic elements of green analytical chemistry approaches, catalysing an interest linked to the development of sustainable paths in research and development activities in the field of viticulture and oenology.

## 1. Introduction

All DIMS techniques are aimed towards directly analysing a complex gaseous mixture using a mass spectrometer. Regardless of the specific technique used, a few common key steps can be highlighted:

(1) Primary ion generation: air, a synthetic gas mixture or a solution are submitted to an electric discharge, microwaves or radiations, which in turn generates a beam or plume of reagent ions.
(2) Ion-molecule reaction: primary ions travel through a drift region where they react with neutral volatile organic compounds (VOCs) contained in the sample. Ionisation proceeds according to one or more mechanisms (e.g., proton transfer, electron capture or hydride abstraction), leading to the formation of product ions.
(3) Analysis: product ions generated during step 2 are further analysed by means of a mass spectrometer.

In order to achieve optimum analytical performance, the generated number of primary ions should be constant and measurable and possibly in large excess with respect to the target molecules that need to be analysed and the ion beam or plume should be of known chemical composition. Ideally, reaction mechanisms and kinetics should be known as well. Under such ideal conditions, high sensitivity and reliable quantification and identification are achieved.

**Figure 1:**
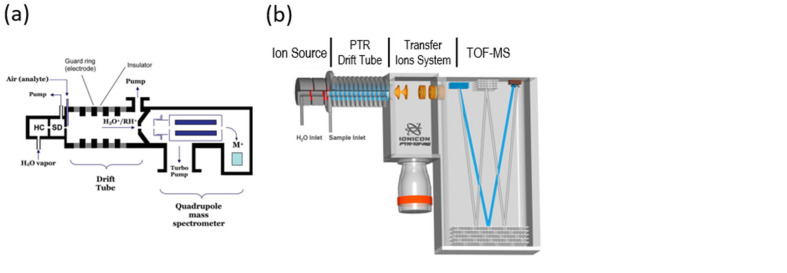

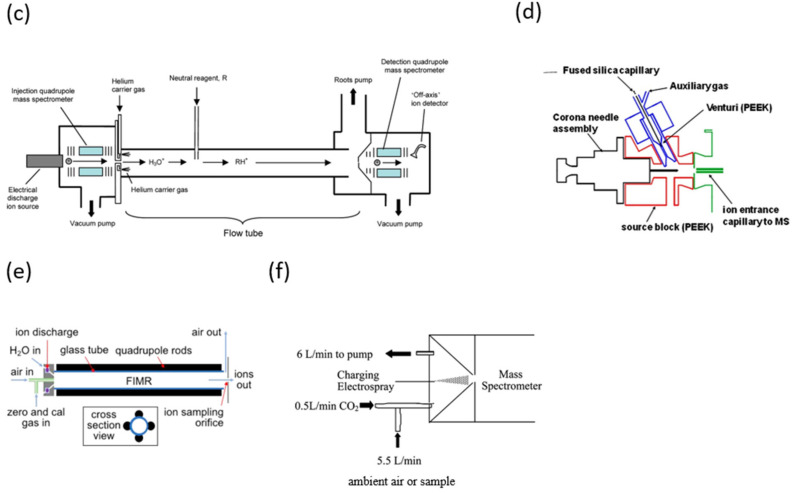
Schematic representation of DIMS instrumentation. (**a**) PTR-MS: reprinted with permission from [6], copyright 2009 American Chemical Society; (**b**) PTR-TOF-MS: adapted with permission from [7], copyright 2009 Elsevier BV; (**c**) SIFT-MS: reprinted with permission from [6], copyright 2009 American Chemical Society; (**d**) APCI source: adapted with permission from [3], copyright 2014 John Wiley and Sons Ltd.; (**e**) VOCUS CI-MS ion source and ion-molecule reactor: reprinted with permission from [4], copyright 2018 American Chemical Society; (**f**) SESI source: adapted with permission from [8], copyright 2008 American Chemical Society.

Over the last four decades, several types of DIMS instrumentation have been introduced as prototypes or commercial instruments. These differ according to the technical solutions which are put in place to achieve ionisation and analysis. A schematic description of DIMS techniques is presented in Table 1, whilst simplified representations of the instruments are provided in Figure 1.

**Table 1:** Main characteristics of DIMS techniques.

Technique	Primary Ion Generation	Ion-Molecule Reaction	Analysis	Ref.
Proton Transfer Reaction—Mass Spectrometry (PTR-MS)	Hollow cathode discharge or α particles	Takes place in drift tube. Ionisation energy depends on electrical field	Quadrupole mass spectrometer: unit mass resolution; isobar and isomer discrimination are not possible	[1]
Proton Transfer Reaction—Time of Flight (PTR-TOF)	Hollow cathode discharge	Takes place in drift tube. Ionisation energy depends on electrical field	Time-of-flight mass spectrometer: accurate mass of analyte ions is determined. Isobar discrimination is possible, isomer discrimination is not possible	[1]
Selected Ion Flow Tube—Mass Spectrometry (SIFT-MS)	Microwave discharge source with quadrupole mass filter	Takes place within flow tube. Product ions transported to mass analyser by means of He/N_2_ flow	Quadrupole mass spectrometer	[2]
Atmospheric Pressure Chemical Ionisation—Mass Spectrometry (APCI-MS)	Corona discharge	The sample enters the source through a venturi inlet, sheathed with a second tube, flushed with a constant flow of nitrogen	Performance depends on mass spectrometer. Most APCI sources are coupled to quadrupole MS	[3]
VOCUS—Chemical Ionisation—Mass Spectrometry (VOCUS-CI-MS)	Discharge reagent ion source	Ion-molecule reactor, consisting of a glass tube with resistive heating, mounted inside a radio frequency quadrupole	Time-of-flight mass spectrometer	[4]
Secondary Electrospray Ionisation—Mass Spectrometry (SESI-MS)	Nano-electrospray ion source	The reagent ions are mixed with the gas carrying the analytes of interest, the charge is then transferred to the analytes	Performance depends on mass spectrometer. Most SESI sources are coupled to Orbitrap MS, which provides accurate mass of analyte ions	[5]

Whereas PTR-MS, SIFT-MS and VOCUS-CI-MS represent all-in-one solutions incorporating a specific type of mass analyser, both APCI and SESI were developed as modular ion sources which can be coupled to different types of mass spectrometers (MSs). It is also worth mentioning that both PTR-MS and SIFT-MS were developed and marketed as quadrupole-based instruments [9]. In a later phase, PTR-MS instruments coupled to time-of-flight (TOF) mass analysers were introduced, both as prototype and commercial versions [7]. The introduction of PTR-TOF-MS, which for the sake of clarity will be henceforth referred to as PTR-TOF, resulted in a remarkable leap forward in analytical capability, with the possibility to measure accurate mass down to the third decimal digit, acquisition speeds of 1 spectrum s^−1^ or lower and a wider mass range. Two different manufacturers produce VOCUS-CI-MS and PTR-TOF-MS, which slightly differ from an engineering standpoint even if the two types of instrumentation are very similar. For a more detailed description of the hardware and mechanisms involved in DIMS analysis, the reader is advised to refer to the relevant literature: a significant, recent reference is reported in Table 1 for each technique.

Regardless of instrument-specific characteristics, with all DIMS techniques, it is possible to analyse the sample directly and online: sample pretreatment is, therefore, minimal or unnecessary. The requirement of chemicals (e.g., derivatising agents) is also minimised. DIMS instruments equipped with time-of-flight (PTR-TOF, VOCUS-CI-MS) or orbitrap mass analysers (SESI-MS) provide data with especially high informational content and reduce the need for the development of specific methods for different classes of target compounds. Based on these elements, and as recently highlighted by a review article [10], DIMS techniques show good compliance with green analytical chemistry principles [11], which advocate for analytical instruments and methods that require fewer harmful chemicals, minimise waste and guarantee improved operator safety.

## 2. DIMS Analysis of Ethanolic Systems

**Figure 2:**
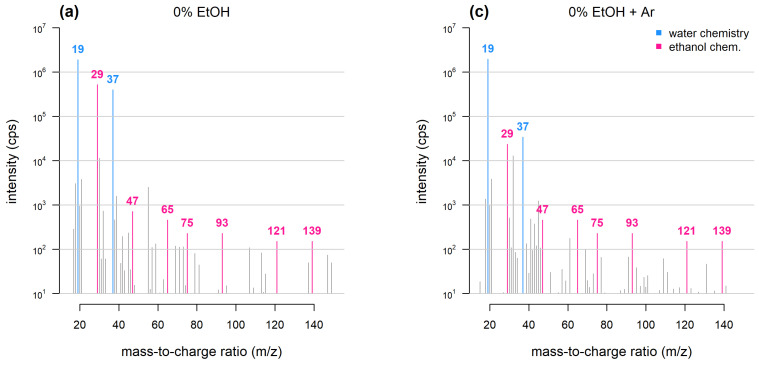

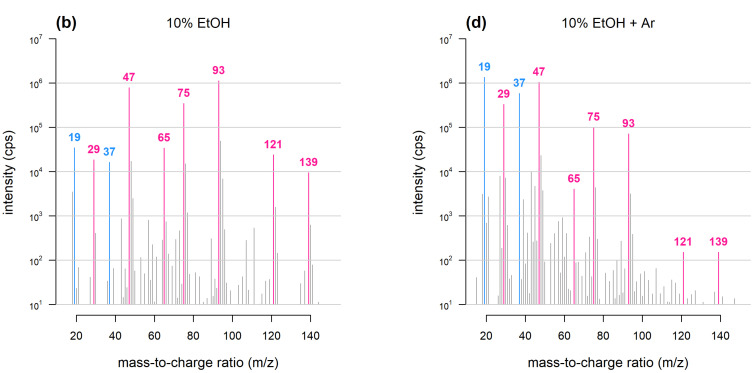
Mass spectra obtained with PTR-TOF. Samples consist of dry air bubbled through water or 10% ethanol (adapted from [16]). Under conventional PTR-TOF chemistry (**a**) and when 10% ethanol is added (**b**), ethanol-related peaks dominate the mass spectrum and deplete water-related peaks. Argon as buffer gas (**c**,**d**) reduces the intensity of ethanol dimers, trimers and ethanol-water cluster peaks and prevents hydronium ion depletion.

A key prerequisite to all DIMS techniques is for the primary ion (in most cases H_3_O^+^) to be largely in excess with respect to the analytes. When ethanol is present at concentrations of at least 2–4% (*v*/*v*) within the sample [12,13], or at least 100 ppmV (parts per million, in volume) within the headspace [14], standard primary ions are nearly entirely depleted, whereas protonated ethanol (*m*/*z* 47), along with other products of ethanol chemistry, act as primary ions. These conditions, which are typically encountered in the DIMS headspace analysis of wine and other alcoholic beverages, result in the following drawbacks:

(1) Since ethanol has a proton affinity (PA) of 776 kJ mol^−1^, which is considerably higher than that of water (PA = 691 kJ mol^−1^), molecules with a PA comprised within that of water and ethanol (e.g., acetaldehyde PA = 710 kJ mol^−1^), can no longer be protonated and are, therefore, not detectable.
(2) Reaction kinetics are more complex because multiple species—each having its own reaction constant and proton affinity—may act as the primary ion. Therefore, mass spectral data are more challenging to interpret. For instance, in the case of PTR-MS analysis conducted under standard conditions ([15] and Figure 2), abundant ions may include protonated ethanol (*m*/*z* 47) water/ethanol clusters (*m*/*z* 65), ethanol dimers and trimers (*m*/*z* 93 and 139) and several dehydration products (*m*/*z* 29, 75 and 121).
(3) Since the amount and type of ethanol-derived ions will depend on how much ethanol is in the headspace, the sample alcohol content will affect the analytical response and sample discrimination may occur mainly based on sample ethanol content.

Over the last two decades, several approaches have been adopted to obviate these problems:

(1) Ethanol saturation: this can be achieved by diluting sample headspace in ethanol-saturated nitrogen when using PTR-MS [14] or by adding ethanol to the makeup gas in APCI-MS [12]. This creates conditions whereby ethanol completely replaces water in driving primary ion generation, and sample ethanol content does not affect results. However, ionisation chemistry remains complex and spectral interpretation is difficult.
(2) Sample dilution: this can be carried out either by diluting the sample headspace in a stream of nitrogen [17] or by adding water directly to the sample prior to analysis [18]. The reduction of ethanol concentration in the sample headspace allows the re-establishment of standard ionisation conditions at the expense of sensitivity.
(3) Argon as buffer gas: PTR-TOF analysis of wine has been carried out by introducing argon into the drift tube either directly or through the sample inlet [16]: this results in a reduction in the proportion of ethanol dimers and clusters (Figure 2). The argon-based approach exploits a previously described effect whereby when air or nitrogen are replaced with a buffer gas having a larger molecular mass (i.e., argon), the internal energy of the reactive species is affected, shifting overall reactivity towards dissociation [19]. However, when argon is injected into the drift tube, water vapour flow needs to be accordingly reduced, resulting in lower sensitivity.
(4) High E/N: the ‘reduced drift field’ (or E/N) is a measure of the energy delivered in ion-molecule collisions. In PTR-MS, E/N is typically 120–150 Td (1 Td = 10^−17^ V·cm^2^). The hydronium ion pool, which is normally depleted measuring ethanolic systems, when operating at conditions of E/N ≥ 250 [20] is replenished thanks to the dissociation of protonated ethanol. On the other hand, high E/N results in high fragmentation and mass spectral data with low informational content.
(5) FastGC-PTR-TOF: a hyphenated system has been reported, coupling PTR-TOF with a short (3.5 m), resistively heated capillary column [21]. FastGC allows to perform a quick (90 s) chromatographic separation prior to analysis: if the first eight seconds of the analysis—during which ethanol is eluted—are discarded, wine headspace analysis can be carried out under conventional PTR-TOF conditions, with the extra benefit of isomer separation.

Among the methods previously cited, ethanol saturation and sample dilution were the first to be applied in 2004 [12,14], whereas the others were introduced later. Some samples of oenological interest, such as grape or cork, can be analysed under conventional DIMS conditions because ethanol is absent from their headspace, or present in small amounts. In other instances, the analysis of ethanolic samples is possible under standard conditions because of the mode of measurement: this happens with nosespace analysis, which is further discussed in Section 6.

## 3. Grape Analysis

DIMS techniques have been applied to grape analysis, effectively addressing oenologically relevant questions and providing information on the quality of raw material.

SIFT-MS has been used to discriminate 23 white and red grape varieties [22]. Key compound classes for discrimination were short-chain alcohols and aldehydes, phenols, monoterpenols and C13-norisoprenoids.

The DIMS approach can also be used to measure the headspace of intact berries throughout the ripening process, thus providing useful indicators of ‘aromatic’ maturity to be used to complement more classic measurements, such as total acidity and sugar content. When SIFT-MS or SESI-MS were used to monitor the ripening of different grape varieties, most mass peaks showed a downward trend, possibly indicative of the fact that volatile compounds are being progressively converted into non-volatile aroma precursors [23,24].

A particular case of the winemaking process is represented by the production of the so-called ‘passito’ wines, whereby grapes undergo withering under controlled conditions. During this process, grapes experience progressive dehydration, accompanied by the development of a typical microflora (i.e., ‘noble rot’). The grape withering characteristic of the production of ‘Amarone’ wine (typical of the Valpolicella region in Italy) has been monitored using PTR-MS [25]. VOC release kinetics allowed us to highlight key processes such as the decline of grape metabolism during dehydration and the release of compounds specific to noble rot (octen-3-one and octen-3-ol).

## 4. Fermentation Monitoring

Alcoholic fermentation (AF) is a process common to all alcoholic beverages. A straightforward AF helps afford a microbially and chemically stable beverage [26], but AF also affects the aroma profile [27]. AF, especially if conducted with industrial starters, takes place in a reasonably short time span, ranging from a few days to a couple of weeks. Therefore, the process lends itself well to online monitoring using DIMS.

The selection of suitable yeast starter strains is a paramount step for every type of alcoholic beverage. PTR-TOF has been employed to monitor VOC production during growth in a synthetic medium by different *Saccharomyces cerevisiae* strains, including two laboratory strains and four wine isolates [28]. The two lab *S. cerevisiae* had an identical genetic makeup with the exception of a single frameshift mutation, affecting amino acid transport. This resulted in measurable differences in the production of higher alcohols (3-methyl-1-butanol, 2-methyl-1-butanol), short-chain fatty acids (isovaleric acid, 2-methylbutanoic acid) and mercaptans (methanethiol). The result exemplifies how DIMS could be used as a high-throughput screening tool in oenological starter selection, but also in metabolomics studies for a better understanding of the biology of model yeasts for wine applications.

DIMS has been employed to study the alcoholic fermentation of mead (i.e., an alcoholic beverage produced from diluted honey). The work [29] compares PTR-MS with Fourier-Transform Infrared (FTIR) spectroscopy and HPLC. Whereas FTIR allows for online process monitoring, HPLC requires offline sampling and analysis and has limited throughput. PTR-MS represents a valuable compromise between the previously mentioned techniques: in fact, it allows for non-invasive monitoring of fermentation kinetics in real time, at the same time providing information on the production or consumption of key metabolites and aroma compounds, such as ethanol, acetaldehyde, acetic acid and esters.

PTR-TOF has been used as an AF online monitoring tool in experimental brewing to investigate the impact of different yeasts and hops and their combination [30]. The results highlighted complex interaction between factors, with hops affecting aroma profile directly, but also indirectly affected by yeast growth and metabolism. Among the VOCs measured, C6 and C8 esters showed complex kinetic profiles with concentration peaks reached at multiple time points throughout fermentation, thus highlighting how high-time-resolution online monitoring can help pinpoint fluctuations that would otherwise be difficult to detect. PTR-TOF was further used to investigate aroma evolution during brewing by studying the impact of different commercial starters [31] and the fate of geraniol contained in hops essential oils [32].

An approach similar to that applied for brewing has been adopted using PTR-TOF to study fermentation and aroma production kinetics during experimental winemaking [33], with AF being carried out using different microbial strains (*S. cerevisiae*, *Metschnikowia pulcherrima* and *Torulaspora delbrueckii* either alone or in combination) in either fresh must or commercial grape juice. In consideration of the different applications, the interest in DIMS techniques to explore the ‘space’ of interactions between the chemistry of the must/wine and the pro-technological microorganisms is evident, as well as in exploring the different interactions between microorganisms of oenological interest (*Saccharomyces* and non-*Saccharomyces* yeasts and malolactic bacteria) that can be inoculated with diverse timing.

## 5. Static and Dynamic Headspace Analysis of Model Ethanolic Systems

During drinking, the first olfactory interaction with the product occurs when aroma compounds reach the nasal epithelium directly through the nose: the process gives rise to the so-called orthonasal perception. The accurate measurement of the composition of the above-the-glass headspace is key to understanding the chemical mechanisms underlying appreciation. In this respect, DIMS techniques, particularly APCI-MS, have been used to characterise the headspace of model solutions mimicking wine or other alcoholic beverages.

In alcoholic beverages, ethanol may affect the air-to-liquid partitioning of aroma compounds in different ways. APCI-MS was used to characterise the headspace composition of water and ethanol/water model systems, showing that in conditions of static equilibrium [12], ethanol reduces headspace VOC concentration: headspace analysis of water or 12% ethanolic solutions containing different VOCs showed a decrease of up to 42% in headspace concentration for the ethanolic system. The impact of ethanol addition on headspace concentration varies as a function of the compound hydrophobicity, as determined by the log *p* value. In fact, the decrease in headspace concentration in the presence of ethanol is directly proportional to the log *p* for log *p* ≤ 3, whereas for log *p* > 3 headspace concentrations are unaffected or even slightly higher in the presence of ethanol. This behaviour is likely related to the different solubilities of each compound in water or ethanol, as well as to the VOC entrapment within micelle-like structures [34].

A major advantage of the DIMS approach is the possibility of directly performing dynamic measurements, which are more representative of real-life/real-system situations. When APCI-MS was used for dynamic headspace measurements in water and in 12% ethanol solutions [35,36], the resulting VOC profiles started with a sharp decrease in measured concentrations, followed by dynamic equilibrium. In ethanolic solutions, VOC concentrations in dynamic equilibrium conditions were generally higher than in water alone; therefore, in such conditions, ethanol helps maintain the compound concentration in the headspace constant and reduces headspace dilution. The magnitude of this effect, which was somewhat affected by the air-to-liquid partitioning coefficient of the target compound, is likely related to the surface-active properties of ethanol. In fact, ethanol evaporation might create local areas of higher evaporation and surface tension, recalling liquid from areas with lower evaporation. The resulting convection phenomenon constantly recalls fresh solution towards the surface, thus helping to keep a constant headspace concentration for most VOCs. The above-mentioned mechanism was later confirmed using thermal imaging [37].

In alcoholic beverages, molecules other than ethanol can also contribute to matrix effects: APCI-MS has been used to study their behaviour. Volatile release has been investigated in model solutions containing odour-active esters and heterocyclic compounds typical of whisky aroma [38]. Ethyl-hexadecanoate, used as representative of long-chain ethyl esters typical of new-make whiskies, reduces aroma release at concentrations above its critical micelle concentration, especially in the case of hydrophobic compounds. This means that whisky dilution may affect perception by altering the balance between hydrophobic and hydrophilic compounds in the headspace, also depending on the content in long-chain ethyl esters. This is particularly relevant in describing real-life conditions, as whisky is often diluted with water to reduce pungency and improve aroma appreciation, both during professional tastings and for everyday consumption. Strecker degradation of amino acids generates odour-active aldehydes which are important in wine and other oenological beverages. The kinetics of the generation of phenylacetaldehyde from its amino acid precursors was studied using PTR-TOF in a model system mimicking Strecker aldehyde generation in wine [39]. The results showed that both glucose and SO_2_ reduce phenylacetaldehyde concentration under wine-like conditions, acting according to different mechanisms. The use of DIMS techniques allows researchers to observe the effect of each reagent addition in real time, with results being generated rapidly and with high confidence.

Another field for the application of DIMS is the direct measurement of carbonated beverages, such as beer or sparkling wine. In such systems, the opening of the bottle creates rapidly evolving dynamic conditions. Therefore, static measurements may provide results which are not representative of the real conditions. When model beers with different compositions were measured using APCI-MS under static equilibrium conditions [40], no statistically significant effect of alcohol, hop acids or carbonation on aroma release was observed. When measurements were repeated immediately after decanting, a significant impact on the release of ethyl-acetate and isoamyl alcohol was observed for all cases. The fact that significant differences are observed only after decanting is relevant as these conditions are closer to actual orthonasal perception immediately prior to—and during—drinking.

GC-olfactometry (GC-O) is another field where standard experimental conditions might not adequately reproduce real-life tasting. In GC-O, an aroma extract, typically obtained by liquid–liquid extraction, is analysed by a trained assessor after chromatographic separation. Since individual compounds are perceived in the absence of matrix effects, this might sometimes result in overemphasised perceived intensity. In a work coupling GC-O and APCI-MS, DIMS was used for its capability to directly measure the headspace of the model system, providing more accurate estimates of perceived orthonasal intensities [41]. This further supported the creation of a dedicated purge and trap system, developed with the aim to collect samples for GC-O.

## 6. Nosespace Analysis

**Figure 3:**
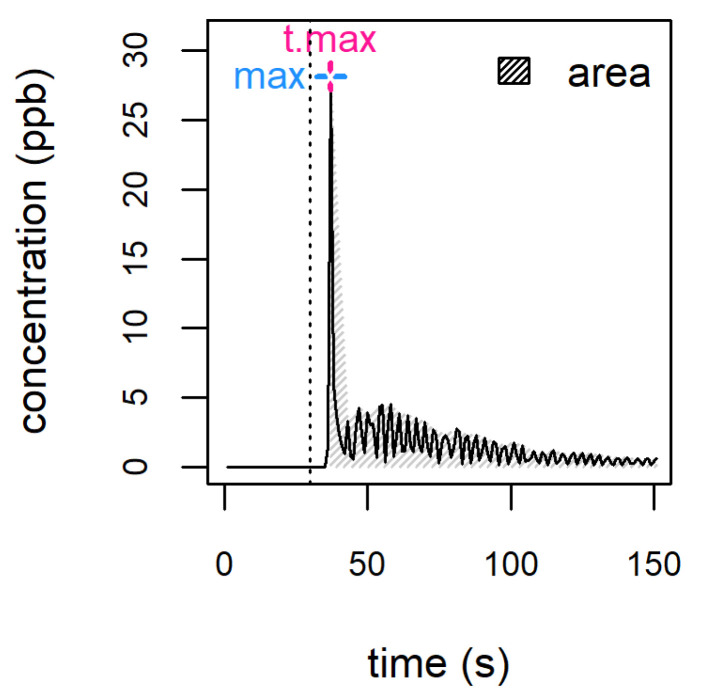
Example of a nosespace profile. Different metrics can be extracted from each profile, such as the area under the curve (area), the maximum intensity (max) and the time after which maximum intensity is reached (t.max). The vertical dotted line marks the moment of sample introduction.

After wine is swallowed, the opening of the epiglottis and the velum–pharynx barrier allows VOCs to travel from the mouth to the olfactory epithelium; in a later phase, VOCs released from liquid remainders adhering to the mouth and throat mucosal surfaces sustain perception over time. This mode of perception is labelled ‘retronasal’ and generally follows orthonasal perception. The possibility of employing DIMS to analyse ’in vivo’ aroma release was first explored by developing a dedicated APCI-MS source [42]. The technique, generally referred to as ‘nosespace’ analysis, is performed by connecting the instrument inlet to the assessor’s nose by means of a custom nosepiece. The results typically obtained consist of time-resolved release profiles (Figure 3); from these, some key metrics of particular significance can be extracted (e.g., ‘area’, ‘maximum’ or ‘t.max’) and used to provide an analytical determination capable of supporting the description of the evolution of perception over time. It is important to note that, due to ethanol sample dilution naturally occurring in the oral and nasal cavities, measurement can be conducted under standard instrumental conditions.

DIMS has been used to perform nosespace measurements on ethanolic model systems [40,43], as well as real alcoholic beverages, including wine [44], brandy [45], vodka [46] and palm wine [47].

When APCI-MS nosespace measurements were performed on model systems mimicking VOC release in beer [40], the maximum and total area of release profiles were increased by both ethanol and CO_2_ addition. In fact, both compounds are surface-active and might increase release directly or by reducing VOC adhesion to mucosal surfaces. The effect of carbonation has also been observed in non-alcoholic beverages during PTR-MS measurements carried out in vivo [48] and using artificial mouths [49]. These findings are of potential relevance for sparkling wines as well, underlining the future potentials of the technique, also in consideration of the relevance of the sparkling wine market.

In more recent works, correlations were sought between taste and VOC release. A model wine mimicking Gewürztraminer was prepared, containing variable amounts of tartaric acid and glucose [50]. The increase in acid concentration in the model wines induced an increased in-mouth release of several compounds (3-methylbutan-1-ol, ethyl-butanoate, isoamyl-acetate and ethyl-hexanoate), as measured by PTR-TOF nosespace. This was in disagreement with headspace measurements, showing that acid or sugar addition did not affect aroma release [51]. However, it must be noted that headspace measurements do not consider saliva and interaction with salivary proteins, which might have affected release in the nosespace experiment. Indeed, experiments conducted using ‘ex vivo’ model systems containing oral mucosa cells [52] show that the addition of mucosal pellicle to the system significantly affected VOC release profiles.

Nosespace experiments conducted on model Gewürztraminer [50] also revealed that if acidity increased, release profiles for ethyl-octanoate decayed faster. In contrast, when sugar increased, the time after swallowing that was required to attain the concentration peak (i.e., ‘t.max’) increased. It is worth noticing that this kind of information concerning intrinsically time-dependent parameters cannot be obtained unless real time measurements are carried out.

In a recent work [44], PTR-TOF nosespace analysis was carried out on model solutions or rosé wines supplemented with different taste and aroma compounds and correlations were sought between oral physiology and VOC release: for many VOCs, total release was negatively correlated with salivary flow and positively correlated with saliva total protein content. Similarly, PTR-TOF online monitoring of an ‘artificial mouth’ system showed that aroma release was increased by adding phenolic compounds in the presence of saliva [53]. These complex interactions observed between oral physiology and VOC release could be explained by considering the dilution and salting out phenomena which are at play during oral processing.

Nosespace analysis also discloses interesting correlations between instrumental DIMS measurements and sensory data. A base wine, modified by oxidation and the addition of oenological tannins was analysed using temporal dominance of sensations (TDS) or PTR-TOF nosespace [54]. An increase in the in-mouth release of short-chain aldehydes (isobutyraldehyde and isovaleraldehyde) might account for an increase in the perception of ‘maderised’ notes and a decrease in the perception of fruity notes observed in oxidised wines. In another study, three commercial samples of baijiu (i.e., a Chinese distillate obtained from different types of grains) were submitted to instrumental and sensory analysis by nosespace VOCUS-PTR-TOF and temporal check-all-that-applies, respectively [55]. VOCs were clustered based on their release profiles: small, hydrophilic compounds characterised early release whereas hydrophobic compounds were typical of late release. Interestingly, key nosespace compounds are different from those highlighted by a previous work based on headspace analysis alone [56].

## 7. Headspace Analysis of Wine and Other Alcoholic Beverages

Whenever a new DIMS-based approach is developed for the analysis of ethanolic systems, wine discrimination has often been employed as a case study. Proof of concept for the PTR-MS ethanol saturation method was first obtained, discriminating commercial red and white wines from different regions and grape varieties [14]. PTR-MS, with the dilution method, was used in the separation of experimental wines obtained from Cabernet Sauvignon and Pinot Noir grapes harvested on different days and at different altitudes. The wine samples from the higher altitude sites and at later harvesting dates displayed higher signal intensities for a number of VOCs as compared to earlier harvested, lowland samples. According to a sensory panel, wines showing high signal intensities also exhibited a higher aroma complexity. A modified dilution method was later used in the discrimination of New Zealand Pinot Noir wines obtained from 6 different vineyard sites [57]. With respect to the method previously used by Spitaler and co-workers [17], a less conservative sample dilution was employed (1:14 vs. 1:40). PCA enabled discrimination of wines based on the vineyard site. Sample separation was due to differences in the ratios of a mixture of compounds, including higher alcohols, ethyl, and acetate esters. Another variation on the dilution method was introduced using PTR-TOF in combination with a liquid calibration unit (LCU) accessory [18]. Samples were diluted 1:20 in water, then vaporised using LCU and directly introduced into the instrument inlet. This allowed the researchers to quantify phenol, cresol and guaiacol responsible for the ‘peaty’ flavour of Scottish whisky.

The PTR-MS high E/N method was tested in the discrimination of eight French brandies from different producers with 2–6 years of ageing [58]. The authors compared results obtained at E/N = 145 and 454 Td. Samples were well separated in both instances, but discrimination took place according to different ions and when the same ions were present in both sets of conditions, they were not likely to come from the same compound. This is not unexpected, as with high E/N, PTR-MS reactivity is definitely shifted more towards dissociation; furthermore, under such conditions, mass spectral interpretation is difficult because multiple analytes may easily give rise to the same fragment.

In the case of APCI-MS, application to real alcoholic beverages has been less frequent, with applications published on the targeted analysis of wort, commercial beer and cider samples [59] and palm wine [60]. PTR-TOF has instead been used more often, possibly due to its improved characteristics in terms of mass and time resolution, guaranteeing improved performance in untargeted analysis. PTR-TOF with argon as buffer gas has been employed in the separation of experimental wines resulting from malolactic fermentation (MLF) performed using three different *Oenococcus oeni* strains [16]. The main drawback when using argon was the reduction in sensitivity (sometimes as high as ten-fold) but the method allowed to preserve standard PTR-TOF chemistry even in the presence of high amounts of headspace ethanol. A difference between pre- and post-MLF samples could easily established based on the production of diacetyl, which is the most abundant MLF-related VOC. Post-MLF wines could also be separated based on the malolactic strain used and discriminating mass peaks were tentatively attributed to other oenologically relevant VOCs such as carbonyls, esters, lactones and alcohols.

The introduction of a FastGC add-on for PTR-TOF represents a further relevant technical innovation, benefiting from the high time resolution of the time-of-flight analyser. FastGC-PTR-TOF was used to analyse 8 different red wines from different region/grape variety combinations [21]. The system also allowed for a direct comparison of dilution and FastGC methods for the same sample. Sample discrimination was possible with both approaches: with dilution, 79 mass peaks were obtained, several of which resulted from abundant wine VOCs such as ethanol, methanol, acetic acid and acetaldehyde. With FastGC, 90 peaks were obtained, which, considering separation, could further be broken down into 132 mass peaks, tentatively assigned to esters, alcohols, terpenes, carboxylic acids, furans, carbonyls, phenols and sulphur compounds. Isomer discrimination and the elimination of ethanol interference were the two main benefits added by FastGC.

Similarly to PTR-MS, ethanol saturation conditions can also be created with PTR-TOF [61]. Through a fine-tuning of instrumental parameters, instrument setup and measurement conditions, it is possible to find ethanol ionisation conditions whereby proton transfer is the main reaction channel. In contrast, product ligand-switching reactions represented about 5% of the total spectrum. Discrimination of freshly distilled and aged French brandies according to region of origin within a well-defined production area (i.e., ‘terroir’) was carried out. Some discriminating peaks could be attributed to known odour-active compounds of French brandies.

## 8. Quality Control

DIMS techniques have been used in quality control of ingredients, materials or finished products. For instance, SIFT-MS has been employed to detect methylamine contamination in agricultural ethanol [13] or to determine methanol content in beer, wine, gin and whisky [62]. Interestingly, the reactivity conditions observed when measuring ethanolic samples with SIFT-MS are close to those occurring in APCI-MS or PTR-MS under ethanol saturation conditions. VOCUS-CI-MS has been used in combination with a dedicated autosampler with the aim to quantify 2,4,6-Thrichloroanisole (TCA), the most widespread cork stopper contaminant [63]. This custom setup allowed the high-throughput analysis of TCA on intact cork stoppers with outstanding results: 5000 stoppers were analysed in a little over 4 h, whereas for a subset of these corks, a good correlation was observed with TCA concentrations obtained according to the ‘releasable TCA’ method [64].

## 9. Twenty Years of Grape, Wine and Alcoholic Beverage Analysis Using DIMS

**Figure 4:**
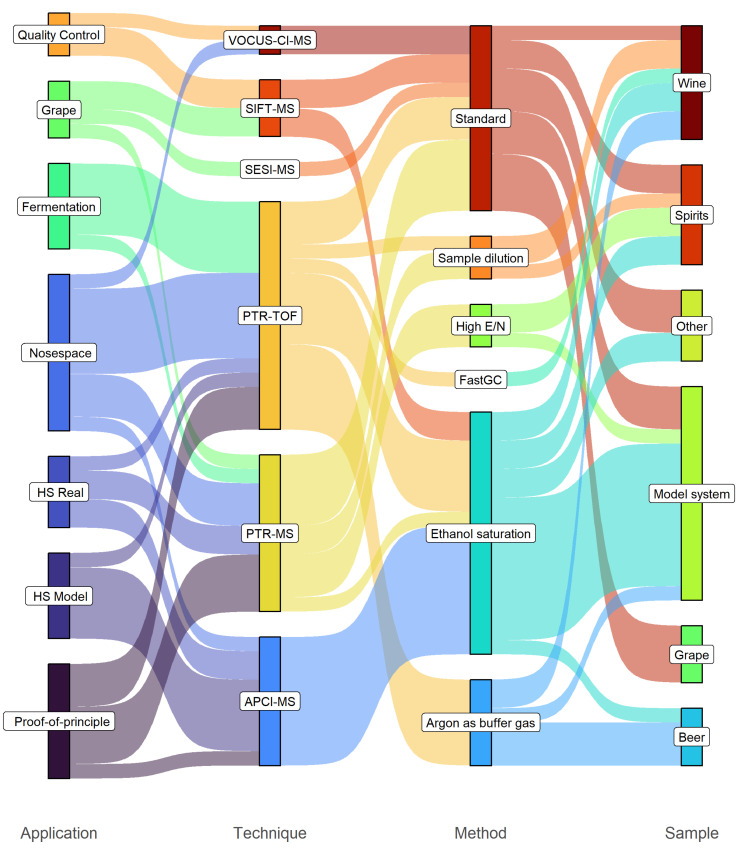
Original articles on DIMS and grape, wine and other alcoholic beverage analysis. The graph shows their distribution in terms of application, technique and method used and type of sample analysed.

The present review covers the application of DIMS to grape, wine, other alcoholic beverages and ethanolic systems in general. Figure 4 shows the distribution of relevant studies based on the type of application, DIMS instrumentation, method employed and samples analysed.

Overall, the progression in complexity of the applications showcased reflects the last two decades of developments in DIMS technology. Among DIMS techniques, APCI-MS was the first to be deployed and marketed. APCI-MS-based applications mainly consist of targeted, static and dynamic headspace analysis of model systems. PTR-MS, and in the following stage PTR-TOF, reflect advancements in terms of standardisation and analytical performance, finally allowing for untargeted analysis of real samples, in high-throughput and time-resolved mode. Out of all the selected papers, PTR-MS and PTR-TOF are the most represented techniques (27 out of 43 reports), followed by APCI-MS (9 reports). SIFT-MS, VOCUS-CI-MS and SESI-MS (4, 2 and 1 reports, respectively) are numerically less represented either because the techniques have more limited diffusion or are of recent introduction. The success of PTR-MS and PTR-TOF can be explained by considering their versatility: as discussed in Section 2, the analysis of ethanolic systems poses specific analytical challenges, which can be overcome by changing the drift voltage, replacing the buffer gas or coupling a FastGC separation step. In fact, even though a FastGC add-on can, in principle, be coupled to any type of DIMS instrumentation, a TOF mass analyser, with its high time resolution, guarantees optimal analytical performance and data quality for this type of hyphenation.

When discussing VOC analysis in wine and other alcoholic beverages, it is important to note that gas chromatography-mass spectrometry (GC-MS) remains the reference technique, as witnessed by the number of published studies. GC-MS is generally more affordable than most DIMS instruments, the know-how relative to the technique is more widespread among scientists and the availability of very large mass spectral databases, especially for mass spectra generated under 70-eV electron impact ionisation conditions, provides an easy-to-access gold standard for compound identification. Indeed, DIMS and GC-MS are not to be seen as competitors but rather as collaborators. The latter has successfully been used to support the former in the case of comparison with reference methods in the quantitation of compounds of oenological interest [63] or to improve compound identification, especially when isomer discrimination is involved [32].

DIMS potentialities are best expressed in applications that exploit its unique characteristics. Section 3 presents applications relative to grape analysis. These exemplify some key advantages of DIMS, such as providing key information on varietal aroma and berry ripening and withering in a completely non-destructive fashion without pre-concentration. Grapes do not have high ethanol content, unlike other types of samples, and analysis is always carried out in standard mode. This review is limited to applications to the grape analysis of strictly oenological interest, but it must be noted that DIMS techniques are also widely employed for fruit headspace analysis and ‘in planta’ VOC measurements: these fields of research deserve to be treated in detail separately and their discussion exceeds the scope of the present review. In any case, this evidence allows us to underline the potential of DIMS from the vineyard to the glass as a kind of ‘sensor’ capable of supporting a holistic approach to the quality of the supply chain through monitoring of volatiles as markers.

Speed of analysis is another clear advantage of DIMS over GC-MS: for PTR-TOF, a throughput of 13–15 samples per hour was reported in the analysis of experimental wines [16]. This makes DIMS especially suited to the analysis of large sample sets. It is also worth mentioning that these high-throughput potentialities are fully exploited only in case DIMS techniques are combined with robotic systems for sample processing (i.e., autosamplers) and suitable workflows supporting the extraction and statistical analysis of large volumes of data [65]. In this case, the potential for exploration of the ‘oenological space’, as influenced by numerous variables such as those linked to the quality of the raw material, the processes, the microbial resources and the chemicals used, demonstrates a potential in offering research and development activities to low cost, can promote innovation in the industry.

The possibility to integrate the time dimension within the analysis is the feature that likely renders DIMS unreplaceable for the characterisation of various processes [66], including those of oenological interest. Dynamic headspace measurements carried out on model systems or real products are of great significance because they help explain time-dependent aroma evolution during tasting by performing measurements in conditions close to those encountered in real life. Moreover, nosespace measures provide a unique window into the tasting process, allowing us to correlate VOC release kinetics with oral physiology and sensory analysis. Finally, DIMS can be used in the time-resolved characterisation of oenologically relevant processes, such as alcoholic fermentation. In this case, collecting multiple time points across a process allows for detailed kinetic characterisation, highlighting key time points that would likely go unnoticed during offline analysis.

The number of DIMS applications of oenological interest is relatively small yet remarkably diverse in terms of instrumental techniques and analytical methodologies used. Still, it is possible to outline some possible future trends. The fact that alcoholic samples are, due to the presence of large quantities of ethanol, not the best suited for DIMS analysis has represented a limitation for the adoption of this approach. On the other hand, this has been—and will be—a driver for developing new, widely applicable instruments or methods, such as FastGC-PTR-TOF or the argon buffer gas method. As a result of the development of multiple analytical strategies, standardisation is poor, and the data available are not easily interoperable. Undoubtedly, it would be of interest to carry out a benchmarking experiment, where a limited number of reference samples (e.g., a small set of experimental wines obtained under controlled conditions) are measured using all the methods cited in Section 2 of this review. In this way, it would eventually be possible to rank all available methods in terms of analytical performance.

Considering the integrated approach, the potential of FastGC coupling to DIMS remains largely unexplored, both within and outside the oenological field. FastGC is as effective as the dilution method in bypassing issues related to ethanol reactivity, it provides a wealth of additional information [21], and it supports mass peak annotation without recurring to cross-platform validation [28]. If properly supported, for example, by developing dedicated retention index libraries or software packages for data analysis, FastGC could become a very valuable weapon of the DIMS analytical armoury.

In conclusion, it is important to underline how DIMS techniques are consistent with various principles of green chemistry, promoting sustainable research and development activities to support the green transition of production systems [10].

## Data Availability

No new data were created or analyzed in this study. Data sharing is not applicable to this article.
